# Antimicrobial Biomaterials for Chronic Wound Care

**DOI:** 10.3390/pharmaceutics15061606

**Published:** 2023-05-28

**Authors:** Adrian Miron, Calin Giurcaneanu, Mara Madalina Mihai, Cristina Beiu, Vlad Mihai Voiculescu, Marius Nicolae Popescu, Elena Soare, Liliana Gabriela Popa

**Affiliations:** 1 Department of General Surgery, Elias Emergency University Hospital, Carol Davila University of Medicine and Pharmacy, No. 37 Dionisie Lupu Str., 030167 Bucharest, Romania; 2 Clinic of General Surgery, Elias Emergency University Hospital, No. 17 Marasti Blvd., 011461 Bucharest, Romania; 3 Department of Oncologic Dermatology, Elias Emergency University Hospital, Carol Davila University of Medicine and Pharmacy, No. 37 Dionisie Lupu Str., 030167 Bucharest, Romania; 4 Clinic of Dermatology, Elias Emergency University Hospital, No. 17 Marasti Blvd., 011461 Bucharest, Romania; 5 Department of Microbiology, Faculty of Biology, ICUB-Research Institute, University of Bucharest, No. 90 Panduri Str., 050663 Bucharest, Romania; 6 Department of Physical and Rehabilitation Medicine, Carol Davila University of Medicine and Pharmacy, No. 37 Dionisie Lupu Str., 030167 Bucharest, Romania; 7 Clinic of Physical and Rehabilitation Medicine, Elias Emergency University Hospital, No. 17 Marasti Blvd., 011461 Bucharest, Romania

**Keywords:** chronic wounds, chronic infections, biofilms, nanotechnology, biomaterials, antimicrobial, biocompatibility

## Abstract

Chronic wounds encompass a myriad of lesions, including venous and arterial leg ulcers, diabetic foot ulcers (DFUs), pressure ulcers, non-healing surgical wounds and others. Despite the etiological differences, chronic wounds share several features at a molecular level. The wound bed is a convenient environment for microbial adherence, colonization and infection, with the initiation of a complex host–microbiome interplay. Chronic wound infections with mono- or poly-microbial biofilms are frequent and their management is challenging due to tolerance and resistance to antimicrobial therapy (systemic antibiotic or antifungal therapy or antiseptic topicals) and to the host’s immune defense mechanisms. The ideal dressing should maintain moisture, allow water and gas permeability, absorb wound exudates, protect against bacteria and other infectious agents, be biocompatible, be non-allergenic, be non-toxic and biodegradable, be easy to use and remove and, last but not least, it should be cost-efficient. Although many wound dressings possess intrinsic antimicrobial properties acting as a barrier to pathogen invasion, adding anti-infectious targeted agents to the wound dressing may increase their efficiency. Antimicrobial biomaterials may represent a potential substitute for systemic treatment of chronic wound infections. In this review, we aim to describe the available types of antimicrobial biomaterials for chronic wound care and discuss the host response and the spectrum of pathophysiologic changes resulting from the contact between biomaterials and host tissues.

## 1. Introduction

By definition, chronic wounds have a slow healing rate and, despite of standard therapy, they lack improvement by 4 weeks and/or persist for more than 12 weeks [1,2]. They tend to recur in over 50% of cases, leading to functional impairment, local and/or systemic complications and decreased quality of life [1,2]. Chronic wounds encompass a myriad of lesions, with a typical clinical picture and evolution due to ischemic, neurotrophic and hypostatic disease such as venous leg ulcers (VLUs), arterial ulcers, diabetic foot ulcers (DFUs) and pressure ulcers, or with atypical traits such as non-healing surgical wounds, ulcers caused by autoimmune disorders, infectious diseases, vasculopathies, neoplasms, metabolic and genetic disorders, external factors, psychiatric disorders, drug-related reactions and others [3,4].

Despite the etiological differences, chronic wounds share several features at a molecular level. They are characterized by an alkaline pH, higher concentrations of matrix metalloproteinases (MMPs), high levels of proinflammatory cytokines, proteases and reactive oxygen species (ROS), deficiency of stem cells and decreased levels of various growth factors, among which are fibroblast growth factor (FGF), epidermal growth factor (EGF), vascular endothelial growth factor (VEGF) and transforming growth factor-β (TGF-β) [2]. Oxidative stress is considered one of the crucial impediments to healing [2,5].

The wound bed is a convenient environment for microbial adherence, colonization and infection, with the initiation of a complex host–microbiome interplay. Mono- or poly-microbial biofilms are complex structures, are very hard to remove mechanically or to treat pharmaceutically and represent the main cause of chronic wound infections [1,6,7]. In acute wounds, microbial infections (mostly bacterial) can be easily treated by topical antiseptics and/or antibiotics. On the other hand, in chronic wounds, microorganisms tend to develop phenotypes of tolerance and resistance to antimicrobials and to the host’s immune defense mechanisms [8,9]. In most cases, patients with chronic wounds also suffer from vascular disease (venous, arterial or both), associated with lower availability of oral therapies to the damaged tissue. Angiogenesis is also impaired by microbial infection, leading to chronic wound hypoxia and inadequate micronutrient or pharmaceutic delivery [10].

Therefore, based on the facts above, systemic antimicrobial therapy is usually not recommended in the treatment of chronic wound infections. On the other side, topical antimicrobials may also fail to achieve bacterial eradication or to prevent recolonization, due to the low penetrance within biofilms or due to the pharmacokinetics and pharmacodynamics of the substances used [6,11].

Wound dressings possess intrinsic antibacterial properties, acting as a barrier to pathogen invasion [12]. Based on the hypothesis that adding an antimicrobial agent to the wound dressing may increase its efficiency to treat infection and accelerate healing, a whole new category of antimicrobial biomaterials has emerged. These may represent potential substitutes for systemic antimicrobial therapy, aiming towards a targeted topical approach against chronic wounds’ colonization and infection.

In this review, we aim to present a detailed description of the available types of antimicrobial biomaterials for chronic wound care and discuss the host response and the spectrum of pathophysiologic changes resulting from the contact between biomaterials and host tissues.

## 2. Classification of Wound Dressings

There are numerous types of wound dressings: traditional ones with cotton gauze impregnated or not with various substances and modern dressings including hydrogels, alginates, film or foam dressings, extracellular matrix (ECM)-derived biomaterials and nanofiber-based dressings, as well as skin substitutes (traditional skin grafts—autografts, allografts or xenografts; tissue-engineered autologous or artificial skin grafts).

Traditional dressings may be impregnated with substances with pro-healing and/or antimicrobial potential such as silver, paraffin, honey, natural plant extracts and others.

Hydrogels are polymeric networks composed of interacting hydrophilic groups that form a three-dimensional matrix [13]. They have the advantage of maintaining moisture, allowing gas diffusion and the absorption of wound exudates [13]. Natural polymers are used to produce hydrogel wound dressings as they are biocompatible, biodegradable and analogous to the extracellular matrix (ECM). Hydrogel alginate-based dressings are hydrophilic, provide a moist wound environment and are highly absorptive if delivered in a lyophilized form. They are highly permeable and non-occlusive; thus, a secondary dressing is needed to stabilize them. Alginate hydrogels can be produced as sheets for surface wounds or as ropes for deep wounds. They can be impregnated with silver or honey for their antimicrobial properties [14].

Film dressings, usually made of polyurethane (PU), are impermeable to liquids and bacteria, simultaneously allowing gas diffusion and providing optimal oxygenation and moisture. However, they have low absorption capacity. The transparency of this type of dressing allows the clinician to visualize the wound, thus monitoring its evolution without removing the dressing [15]. They are not recommended for the treatment of highly infected wounds and in areas where the skin is sensitive/fragile, as tissue trauma may ensue upon removing the dressing [14].

Foam dressings have excellent absorbing properties. They are made of a porous material consisting of PU or polyvinyl alcohol (PVA) and do not macerate the tissue; thus, they may be changed less often [16]. PU-absorptive foam dressings have a hydrophilic surface placed at the interface with the wound. The hydrophobic surface is in contact with the outside environment. They are permeable to gas but not bacteria and other pathogens. The bacteriostatic effect of foam dressings impregnated with methylene blue was proven by several studies [14].

ECM-Derived Biomaterials represent a network of macromolecules synthesized by cells, such as epithelial cells, fibroblasts and mesenchymal cells, forming a skeleton-like structure. Natural tissues can be decellularized by physical, chemical and enzymatic methods, resulting in a decellularized extracellular matrix (dECM). An acellular matrix is obtained by removing the cellular components of a tissue or organ but keeping the structure and fibrillar components. It is a highly biocompatible and nonimmunogenic natural scaffold. dECM hydrogel retains a number of cell growth factors, such as FGF, TGF and hepatocyte growth factor, which can enhance the growth, proliferation, differentiation and angiogenesis of the seed cells. ECM materials and scaffolds produced from decellularized tissues and organs are valuable therapeutic alternatives in wound healing since acellular ECM and ECM-based scaffolds have the ability to stimulate natural tissue regeneration [17,18]. Some of the clinical applications of dECM include soft tissue repair (burn wounds, diabetic foot ulcers, etc.), in which products derived from decellularized human skin, porcine small intestine submucosa and the porcine urinary bladder are used [19]. By additional processing techniques, dECM may present as hydrogel, powder or a second sheet [18]. 

Nanofiber-based dressings have a high surface-to-volume ratio and a structure similar to the ECM. Cellulose is a fibrillar biomaterial. Still, other natural biomaterials, such as chitosan, silk fibrinoid and collagen, may be used to prepare nanofibers by various techniques [13].

Skin substitutes are traditionally known as skin grafts, including autografts (taken from the patients), allografts (taken from cadavers) or xenografts (harvested from animals—pigs, fish or others). New skin substitutes are represented by tissue-engineered autologous or artificial skin grafts. Based on the anatomy of the skin, tissue-engineered autologous skin grafts can be classified as epidermal, split-thickness and full-thickness [20,21]. Recent trends point towards the development of nanocomposite skin substitutes with controlled antimicrobial activity [22].

## 3. Biomaterials and Antimicrobial Agents for Chronic Wound Care

Biomaterials are available as films, foams, hydrocolloids, hydrogels, scaffolds (electrospun and 3D-printed), hydro-fibers and the recently introduced electrospun nanofibers [23]. They can be divided into two major categories: natural and synthetic. Natural biomaterials include biological products originating from plants, fungi, bacteria or animals and can also be divided into polysaccharides, proteins and macromolecules combining both. Matrices obtained from decellularized tissues are also considered natural materials [15]. Synthetic biomaterials are represented by organic and inorganic polymers.

While some biopolymers, such as chitosan, have shown inherent bactericidal properties, other polymeric wound dressings must be supplied with antibacterial agents to prevent infection [24].

The production of synthetic polymers is easier and cheaper. Some, such as PU, are nonbiodegradable, while others, such as polycaprolactone (PCL), PVA, polyglycolic acid (PGA) and poly d,l-lactide-co-glycolide (PLGA), are biodegradable. This category of biomaterials does not possess inherent antibacterial effects. They may, however, be used in blends with natural polymers, such as chitosan [25].

In contrast to synthetic polymers, polymers having a natural origin (cellulose, starch, chitosan, alginate, etc.) are preferred for biomedical applications because of their higher biocompatibility and biodegradability and because of their low-cost production [26].

**Figure 1:**
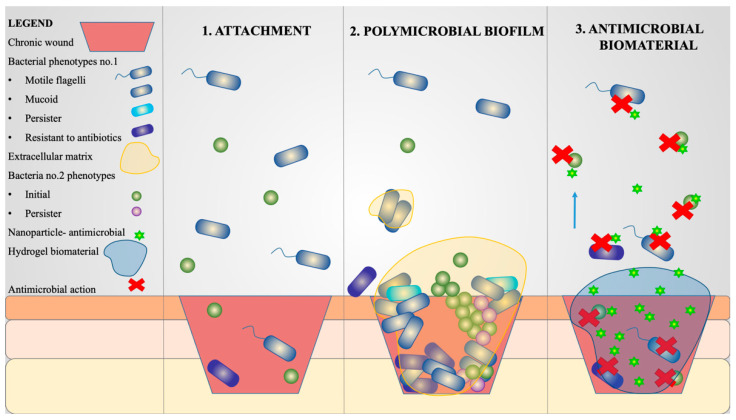
Bacterial species with different virulence and antibiotic resistance phenotypes (motile and resistant to antibiotics) may attach to the chronic wound’s bed and develop mono- or poly-microbial biofilms. Within biofilms, persister bacteria show tolerance to antimicrobials. Biomaterials impregnated with antimicrobials (e.g., antibiotics, metal nanoparticles, hybrid nanoparticles) impede the attachment of bacteria and release therapeutic agents, destroying free-floating microorganisms.

Biomaterials can be loaded with antimicrobial agents (antibiotics, antimicrobial nanoparticles, cationic organic agents and others) to prevent wound colonization and treat infection (Figure 1) [27].

Section 3.1 summarizes some natural polymeric biomaterials that can be or have already been included in dressings, together with antimicrobial and antioxidative agents (described in Section 3.2–3.4), to develop an ideal dressing that maintains moisture, allows water and gas permeability, absorbs wound exudates, protects against bacteria and other infectious agents, is biocompatible, non-allergenic, non-toxic, biodegradable, easy to use, easy to remove and, last but not least, is cost-efficient (Table 1) [15].

### 3.1. Natural Polymeric Biomaterials

Alginate, a marine biopolymer, is a natural polysaccharide extracted from the cell walls of brown algae. Its structure consists of a linear copolymer of α-L-glucuronate and β-D-mannuronate, covalently linked in various arrangements. The physical properties of alginates are influenced by the proportion of these arrangements. Sodium and calcium alginates are the two main categories. The most common production method for an alginate dressing is cross-linking of the sodium alginate solution with divalent ions (calcium, magnesium, zinc, etc.) in order to obtain a gel. The gel can be further processed to produce a foam-like or fibrous dressing [16].

Alginate dressings effectively reduce the inflammatory phase of a wound, resulting in improved wound healing. Alginate and its compounds manifest absorbent properties (ideal to manage large amounts of exudate) and show high stability in maintaining structural integrity [28].

Upon contact of the alginate dressing with the wound exudate, an ion exchange takes place, involving the calcium ions in the dressing and the sodium ions in the blood and wound exudate. When sodium ions replace sufficient calcium ions, the alginate fibers swell and transform into a gel that ensures moisture and painless changes of the wound dressing [29].

Cellulose is the main component of plants, algae, fungi and bacteria. It is a polysaccharide that contains a linear chain of several hundred to many thousands of β (1 → 4)-linked D-glucose units. Bacterial cellulose (BC) has numerous properties essential for wound healing materials (water retention capacity and mechanical strength) [15]. BC itself lacks antibacterial properties and does not provide a barrier against infection [15]. Using biotechnology, BC can be modified to enhance solubility and antibacterial potential and decrease immunogenicity. As an example, Ahamed M.I.N. et al. (2015) designed a cellulose–chitosan hybrid nanocomposite containing a mixture of silver nanoparticles (AgNPs) and gentamicin for wound dressing applications [30]. Additionally, Petrova et al. (2020) observed that the impregnation of BC with a solution of polysaccharides–hyaluronan, sodium alginate, κ-carrageenan and chitosan leads to the formation of a new composite, with a better compatibility with mesenchymal stem cells, whose properties can be influenced by changing the concentrations of the previously mentioned substances [31]. Asanarong et al. (2021) observed that immersing BC-glutaraldehyde in papain leads to the formation of a superior biocomposite, both from the point of view of mechanical properties, and from the point of view of the in vitro inhibition capacity of some microorganisms frequently involved in the superinfection of skin wounds (*P. aeruginosa*, *Escherichia coli—E. coli*, *S. aureus—S. aureus*) [32].

Montmorillonite (MMT) is a commonly used, non-toxic medicinal clay, and a phyllosilicate based on two tetrahedral sheets of silica sandwiching a central octahedral sheet of alumina spaced out interlayer spaces (galleries) [33]. Its products are used for their protective and antibacterial properties. Studies have shown that MMT and its products can adsorb bacteria such as *E. coli* and *S. aureus*. A modified MMT has been synthesized by adding inorganic elements, such as silver and copper. Composites of BC with Na-MMT, Ca-MMT and Cu-MMT have been prepared to pass on antibacterial activities to the BC sheets. Ul-Islam M. et al. (2013) showed that BC-Cu-MMT composites are highly efficient against *E. coli* and *S. aureus* [34]. Moreover, the antibacterial effects were more remarkable with higher MMT concentrations [34]. García-Villén F. et al. (2019) showed that compared to the free drug, a clay mineral–drug nanocomposite based on montmorillonite and norfloxacin showed superior antimicrobial activity against *P. aeruginosa* and *S. aureus* [33].

Chitosan (Ch) is a linear polysaccharide derived from chitin through deacetylation. Nature abounds in chitin, and it is primarily obtained from the exoskeletons of crustaceans and insects and from fungi. Chitosan contains amino groups; therefore, it is the only natural polysaccharide that is positively charged [15]. It interacts with negatively charged species such as glycosaminoglycans (GAGs) and red blood cells. Chitosan is an antibacterial, antifungal, mucoadhesive, analgesic and hemostatic biomaterial [15]. It undergoes enzymatic degradation by the lysozyme. The degradation products of chitosan, glucosamine and N-acetylglucosamine, are naturally occurring molecules in the human body used to synthesize GAG. The biocompatibility of chitosan is directly proportional to the glucosamine units [15]. In order to slow down its degradation rate, chitosan is cross-linked.

Chitosan can be processed into functional dressings, such as films, fibers, sponges and hydrogels [16]. It may be applied as coated-fabric materials or combined with different polymers such as PVA and PEG. Chitosan accelerates wound healing and stimulates the immune response due to its hemostatic, antibacterial and non-toxic properties [35]. All these features make this natural polymer an outstanding candidate for tissue engineering [36].

Ch-AgNPs showed effective antibacterial action and control over methicillin-resistant *S. aureus* (MRSA) and *Pseudomonas aeruginosa* (*P. aeruginosa*) bacterial biofilms [37]. The biofilms formed in the presence of Ch-AgNPs detached more easily with surfactant than those without Ch-AgNPs [37]. In addition, Ch-AgNP-coated clinical band-aid cloth showed enhanced antibacterial activity against MRSA and P. aeruginosa [37].

Chitosan microspheres loaded with silver sulfadiazine encapsulated in PEGylated fibrin gels also exhibited intense antimicrobial activity against *S. aureus and P. aeruginosa* [38].

Other biopolymers are fucoidan, hyaluronic acid and collagen. They promote wound healing and are well tolerated due to their characteristics: non-toxic, biodegradable, biocompatible and non-immunogenic [39]. Nevertheless, collagen-based materials are often permeable to external pathogens [16].

### 3.2. Nanomaterials—Inorganic Antimicrobial Nanoparticles Incorporated in Biomaterials

Metal and metal oxide nanoparticles (NPs) such as gold, silver and zinc oxide are active at low concentrations against various infectious agents. NPs modulate microbial colonization and biofilm formation in wounds [7].

The physical and chemical features of metal NPs are essential for their antimicrobial and antibiofilm activity. Metals have excellent antimicrobial activity, but their physicochemical properties change greatly from the bulk material to the nanoscale due to size and shape effects and the high surface area to volume ratio of nanomaterials [40].

Nanotechnology is based upon engineering substances at the nanoscale, modifying their physical and chemical properties. NPs are structures with dimensions ranging from 1 to 100 nm that exist as a total unit. They can be either of organic or inorganic origin [41].

Compared to conventional biomaterials, nanoparticle-based therapies used in treating wounds are relatively new. Silver NP-based creams/ointments are widely used primarily because of the antimicrobial properties of nanocrystalline silver [14]. 

Nanotherapies can be effective at lower concentrations because they have a high surface-to-volume ratio. Many materials exhibit greater antibacterial properties at the nanoscale compared with their original form. Metals with intrinsic antimicrobial properties (silver, copper and zinc) have broad-spectrum antimicrobial effects, but they tend to aggregate in the absence of any material support. Silver and zinc NPs have been used as microbicidal substances in plant-based dermatological preparations. Biosynthesized silver NPs are reported to exhibit exceptional antibacterial activity against a wide range of microorganisms, as well as antifungal activity [40].

Positively charged NPs exert their bactericidal effect by releasing toxic metal ions or ROS. When in direct contact with bacteria, the positively charged groups bind to the bacterial surface negatively charged groups, creating “holes” in the bacteria and allowing NPs to penetrate the cell wall and disturb the metabolic pathways [42].

Several studies have examined the effects of chitosan hydrogels associated with nano-Ag and AgNPs [42]. In an in vitro study, Wu J. et al. (2014) showed that wound dressings composed of spherical AgNPs of 10–30 nm incorporated in nanofibers lowered the *E. coli*, *S. aureus* and *P. aeruginosa* counts by more than 99% [42,43].

The toxicity of NPs depends on their structure and configuration. In addition, the same type of NPs may have a different effect depending on the bacterial species involved. CuONPs and ZnONPs are very efficient against *E. coli*, but less efficient against *S. aureus* and *Bacillus subtilis (B. subtilis)*. AgNPs also exert a better antimicrobial effect on *E. coli* and *S. aureus* compared to CuNPs. In addition, CuONPs are more toxic [42].

Silver nanoparticles (AgNPs) are used as an antimicrobial agent in the treatment of venous ulcers/burns, given their activity against antibiotic-resistant bacterial strains. Several mechanisms of action have been proposed, among which are the alteration of the mitochondrial respiratory chain, the release of large amounts of superoxides and nano-silver ions and the destruction of the nucleus and genetic material [44]. Silver nanoparticles can also interfere with anti-inflammatory cytokine release, thus promoting wound closure [13]. To date, no reports suggest the development of resistance when used for clinical applications. AgNPs target pathogens and human eukaryotic cells at a specific concentration, but only microorganisms are damaged [44]. Ag displays a concentration-dependent cytotoxic effect on human dermal fibroblast cells. Using nanotechnology, specialists established a therapeutic window that enhances the antimicrobial properties of Ag and decreases its minimum inhibitory concentration, as well as reducing toxicity to human cells [42]. 

Several clinical trials have been conducted using commercially available AgNPs and found that they do not have a clinically significant change in human metabolic, hematologic or urinalysis profiles. Moreover, no morphological changes were observed in critical human organs, including the lungs and heart, and no changes in pulmonary ROS formation or pro-inflammatory cytokine production were noted [45].

Zinc oxide nanoparticles (ZnONPs) possess antimicrobial properties, the main mechanism of action being the production and release of ROS, leading to protein and DNA damage and cell death. ZnO promotes wound healing due to its anti-inflammatory effect [1]. The beneficial effect on re-epithelization via keratinocyte migration has been demonstrated when incorporated into hydrogel-based dressings [13]. Ahmed S. et al. (2015) synthesized chitosan composites enriched with different concentrations of ZnO [46]. The composites exhibited a significantly lower degradation rate and higher thermal stability than chitosan alone and exerted biocidal effects on Gram-positive and Gram-negative bacteria [46].

Gold nanoparticles (AuNPs) exert bactericidal and bacteriostatic properties due to their ability to bind to the bacterial DNA and directly target the bacterial cell wall [13]. AuNPs are biocompatible; therefore, they are employed mainly in biomedical domains. AuNPs may be combined with gelatin, chitosan and collagen to treat wounds. Some studies indicate AuNPs have low or absent cytotoxicity, and when present, it is related to the nanoparticle’s size, shape and surface charge. It has been suggested that AuNPs are safer on mammalian cells than other NPs because their mechanism of action is not ROS-dependent [47].

Titanium dioxide NPs (TiO2NPs) have long been considered to exert their antimicrobial activity through the release of ROS, the oxidative stress leading to DNA damage [47]. Roy et al. (2010) studied the effect of TiO2NPs in combination with different antibiotics against *MRSA* and suggested that TiO2NPs not only improve the antimicrobial effect of various antibiotics against *MRSA* but also decrease the risk of antimicrobial resistance of *MRSA* against different antibiotics [48]. Archana et al. (2013) showed that wound dressings loaded with TiO2NPs and coated with chitosan and pectin are antimicrobial and have wound healing properties [49].

Silica nanoparticles (SiO2NPs) are very effective against *P. aeruginosa* and *E. coli*. Besides their antibacterial properties, they are beneficial in wound healing due to their ability to inhibit the rate of fibroblast proliferation. SiO2NPs also inhibit bacteria adherence to oral biofilms [47].

Magnesium oxide NPs (MgONPs) cause injuries to the bacterial cell membrane and subsequent leakage of the cellular components, resulting in cell death. A study by Sawai et al. (2000) on the effects of MgO on *E. coli* and *S. aureus* concluded that the main determinant of MgONP antimicrobial activity is the presence of ROS on their surface [50]. Nonetheless, it was later observed that MgONPs exert strong antibacterial activity even in the absence of ROS production [47]. Liu et al. (2023) also observed that the incorporation of MgO nanoparticles on nanofibrous membranes leads to the formation of biomaterials whose properties are proportional to the amount of MgO incorporated—a relationship of direct proportionality with bacterial inhibition, but of inverse proportionality with regard to the stimulation of cells that promote healing: fibroblasts, endothelial cells and dendritic cells [51]. The same authors highlighted the usefulness of the same membranes loaded with MgO nanoparticles in promoting angiogenesis (by inducing vascular growth factors), in decreasing the immune response and in favoring the formation of granulation tissue at the level of the wound in rat models, with promising results to be applied to diabetic patients [52].

### 3.3. Nanomaterials—Organic Antimicrobial Nanoparticles Incorporated in Biomaterials

Organic NPs such as liposomes, micelles and aptamers may also be included in biomaterials for a targeted drug delivery within wounds [53,54,55,56]. Using liposomes, the drugs (antibiotics and antifungals) may be released straight into the cells, with a diminished toxicity and increased anti-biofilm efficacy and biocompatibility [53,55,57]. Moreover, antimicrobials can be released for longer periods of time to obtain a sustained treatment for wound infection. 

As an example, Wang S. et al. (2021) developed double network hydrogels based on liposome, polyethylene glycol (PEG), α-cyclodextrin (α-CD) and acrylamide (AM) (PEG-α-CD/AM/liposome @amoxicillin double network hydrogels) [58]. The liposome acts as an amoxicillin repository, allowing for a sustained drug release of up to 12 days [58].

### 3.4. Plant-Derived Composites Incorporated in Biomaterials

Plant-derived composites have been used to modify biomaterials for the treatment of chronic wounds. By adding vitamins or drugs, biomaterials have superior biological properties and biocompatibility [16].

Non-conventional methods have been used to treat chronic wounds, including plant extracts, essential oils and honey because of their antioxidant, anti-inflammatory and antibacterial properties. They have been formulated as hydrogels, films, foams or sponges [26]. So far, the electrospinning method has shown promising results in the production of advanced drug delivery systems compared to other methods, such as physical or chemical adsorption [16].

Plants are an essential natural resource with multiple therapeutic activities, including antimicrobial effects, and research in the last decade has taken them into account for inclusion in biomaterials for wound treatment.

Curcumin, a phytochemical component of spice turmeric (*Curcuma longa*), has antioxidant, hypoglycemic, anti-inflammatory, anti-rheumatic and antibacterial activity. In addition, it is non-toxic and has high biocompatibility [16]. The inclusion of Ag, Au, Cu or Zn NPs in various biomaterials with antimicrobial properties, such as hybrid nanocomposites, carbon nanocomposites, antimicrobial hydrogels and chitosan-based nanomaterials, was studied. The antibacterial activity against *E. coli* was enhanced by adding curcumin into a Ch-PVA/Ag nanocomposite hydrogel [26].

Abbas et al. (2019) also produced a chitosan/PVA/curcumin membrane with variable content of curcumin and chitosan [59]. The most significant reduction in wound size in a rabbit model was obtained for the biomaterial containing the highest concentration of curcumin [59]. The same biomaterial also showed antibacterial (*Pasteurella multocida*, *S. aureus*, *E. coli* and *Bacillus subtilis*) and antioxidant properties [59]. Furthermore, the effect of curcumin encapsulated into a saline–hydrogel nanoparticle vector was investigated. Curcumin displayed in vitro antibacterial activity against *MRSA* [13,59].

A Chinese patent studied the production by electrospinning of a bioactive dressing of chitosan and curcumin that showed anti-inflammatory properties and antibacterial activity against *E. coli* [16].

Another study in which thyme oil was incorporated in chitosan at a concentration of 1.2% showed an antimicrobial effect against *E. coli*, *Klebsiella pneumoniae*, *P. aeruginosa* and *S. aureus* [60].

Chen Y. et al. (2020) also analyzed the antibacterial properties of thymol’s addition to cellulose fibrous membranes and concluded that the porous biomaterial with the highest thymol content (15%) had the best antibacterial activity (bacteria survival rate equal to 0.07% for *S. aureus* and 0.09% for *E. coli*) [61].

Cinnamon oil was used by Qin M. et al. (2021) for in situ fabrication and application of wound dressings. The biomaterial showed strong antibacterial activity, with the inhibition zones of *E. coli* and *S. aureus* exceeding 5 cm. In the absence of cinnamon oil, the biomaterial had weak antibacterial activity [62].

Honey has well-known antimicrobial, anti-inflammatory and antioxidant effects and promotes granulation and re-epithelialization through angiogenesis [13]. Its antibacterial properties are given by glucose–oxidase, which enables the formation of hydrogen peroxide in the wound bed, which has an antiseptic effect and inhibits biofilm formation [16]. Its low pH allows the release of oxygen from hemoglobin, creating an unfriendly environment for bacteria. Additionally, its high content of glucose, fructose and polyphenols aids in the process of wound healing. Manuka honey, obtained from the nectar of *Leptospermum scoparium* is the honey type most commonly used in wound dressings, given its proven antibacterial activity against *S. aureus*, *P. aeruginosa* and vancomycin-resistant enterococci [15]. Honey-impregnated dressings are used for leg ulcers, burns, surgical wounds, boils, etc. It possesses excellent biocompatibility because of its natural origin, but it is contraindicated in patients with a bee venom allergy or honey allergy history [15]. Polysucrose-based hydrogels may be loaded with Manuka honey, *Eucalyptus* honey or other natural molecules/extracts such as *Gingko biloba*, thymol, metformin, etc. [63].

**Table 1:** Biomaterials and antimicrobial agents for chronic wound care. Abbreviations: Montmorillonite—MMT, *Stapylococcus aureus*—*S. aureus*, Methicillin-resistant *S. aureus*—MRSA, *Pseudomonas aeruginosa*—*P. aeruginosa*, *Escherichia coli*—*E. coli*, Nanoparticles—NPs.

No.	Name	Short Description	Antimicrobial Properties	Ref.
A. Natural polymeric biomaterials				
1.	Alginate (sodium alginate and calcium alginate)	natural polysaccharide linear copolymer of α-L-glucuronate and β-D-mannuronate, covalently linked in various arrangements hydrogel, film, foam and fibrous dressings	No intrinsic antimicrobial activity.	[16,39]
2.	Cellulose (bacterial cellulose and others)	natural polysaccharide linear chain of several hundred to many thousands of β (1 → 4)-linked D-glucose units hydrogel, hydrocolloid, film, foam and sponge	No intrinsic antimicrobial activity.	[15,64]
3.	Montmorillonite (MMT)	non-toxic medicinal clay phyllosilicate based on two tetrahedral sheets of silica sandwiching a central octahedral sheet of alumina spaced out interlayer spaces (galleries)	No intrinsic antimicrobial activity.	[34]
4.	Chitosan	the only natural polysaccharide that is positively charged (due to amino groups) derived from chitin through deacetylation films, fibers, sponges and hydrogels	Intrinsic antimicrobial activity: bactericidal and fungicidal.	[15,16,24]
5.	Other biopolymers: fucoidan, hyaluronic acid and collagen	non-toxic, biodegradable, biocompatible and non-immunogenic	No intrinsic antimicrobial activity. Permeable to pathogens.	[16,39]
B.	Inorganic antimicrobial nanoparticles (metal and metal oxide NPs) incorporated in biomaterials	structures with dimensions ranging from 1 to 100 nm metals and metal oxide NPs with intrinsic antimicrobial properties:	Intrinsic wide antimicrobial activity.	
		silver—AgNPs copper—CuONPs zinc oxide—ZnONPs gold—AuNPs titanium dioxide—TiO2NPs silicon dioxide—SiO2NPs magnesium oxide—MgONPs		
C.	Organic antimicrobial nanoparticles incorporated in biomaterials	liposomes, micelles and aptamers releasing the drug (antibiotics, antifungals and cytotoxic drugs or vaccines)	They release antimicrobial drugs.	[53,55,57]
D.	Plant-derived composites incorporated in biomaterials	non-toxic and high biocompatibility	Intrinsic antimicrobial activity.	[16,62,63]
		curcumin thyme oil cinnamon oil Manuka honey and Eucalyptus honey or other natural molecules/extracts		
E. Antimicrobial biomaterials—examples of composites cited in this article				
1		cellulose–chitosan hybrid nanocomposite with AgNPs and gentamicin	Antimicrobial	[30]
2		bacterial cellulose-copper—MMT composite	*E. coli* and *S. aureus*	[34]
3		chitosan—AgNPs	MRSA and *P. aeruginosa*	[37]
4		chitosan microspheres loaded with silver sulfadiazine encapsulated in PEGylated fibrin gels	*S. aureus* and *P. aeruginosa*	[38]
5		spherical AgNPs of 10–30 nm incorporated in bacterial cellulose nanofibers	*E. coli*, *S. aureus* and *P. aeruginosa*	[43]
6		chitosan composites enriched with different concentrations of ZnO	Biocidal effects on Gram-positive and Gram-negative bacteria	[46]
7		wound dressings loaded with TiO2NPs and coated with chitosan and pectin	Antimicrobial	[49]
8		curcumin—chitosan-PVA/Ag nanocomposite hydrogel	*E. coli*	[26]
9		chitosan/PVA/curcumin membrane with variable content of curcumin and chitosan	*Pasteurella multocida*, *S. aureus*, *E. coli* and *Bacillus subtilis*	[59]
10		curcumin encapsulated into a saline–hydrogel nanoparticle vector	MRSA	[13,59]
11		electrospun bioactive dressing of chitosan and curcumin	*E. coli*	[16]
12		1.2% thyme oil incorporated in chitosan	*E. coli*, *Klebsiella pneumoniae*, *P. aeruginosa* and *S. aureus*	[60]
13		cellulose fibrous membranes enriched with different concentrations of thymol	*S. aureus* and *E. coli*	[61]
14		cinnamon oil in alginate gel	*E. coli* and *S. aureus*	[62]

## 4. Host Immune Response to Implanted Antibacterial Biomaterials 

The implantation of biomaterials generates various degrees of tissue injury, depending on their nature and the procedure employed. This triggers a host response that attempts to restore homeostasis. As a result of the biomaterial’s contact with blood and tissues, a spectrum of pathophysiologic changes ensues. These include the sequential or overlapping development of acute and chronic inflammation, followed by the formation of granulation tissue, a foreign body reaction and eventually fibrosis. The amplitude and duration of the host reactions define the biocompatibility of a certain biomaterial, which is influenced by its size, shape and chemical and physical characteristics [65].

### 4.1. Provisional and Final Matrix Formation

The host’s initial response to the tissue trauma and vascular disturbance caused by the implantation of a biomaterial consists of inflammation, with vasodilatation, increased vascular permeability, exudation of plasma and blood cells into the injured tissue, thrombosis and recruitment and activation of inflammatory cells [65].

Minutes to hours after implantation, the biomaterial surface becomes the site of thrombosis and provisional matrix formation [65]. The latter consists of the deposition of a protein film composed of coagulation system components (fibrin, fibrinogen and high molecular weight kininogen), complement fragments (C3 and C5) and other blood proteins [especially albumin, immunoglobulin (Ig) G, fibronectin and vitronectin], as well as activated platelets and endothelial and inflammatory cells [65,66,67]. The protein network ensures structural support, facilitates cell adhesion and migration and modulates cellular responses. In addition, effective elements such as mitogens, chemoattractants, cytokines and growth factors are constantly released from the provisional matrix, influencing the activity and interactions of various cells implicated in tissue repair and wound healing [65,67].

Subsequently, a final matrix develops at the surface of the biomaterial, composed predominantly of fibrin. A fraction of the fibrinogen adherent to the biomaterial surface is not cleaved to fibrin and interacts through its functional epitopes with integrin receptors on the surface of platelets, neutrophils and monocytes [66].

### 4.2. Acute Inflammation

During the acute phase of the inflammatory response, which may last hours or days, depending on the severity of tissue injury, neutrophils infiltrate the site. They are attracted to the implantation site by danger signals released as a consequence of tissue injury. These are referred to as damage-associated molecular patterns (DAMPs or alarmins) and include adenosine triphosphate (ATP), uric acid, bioactive lipids and heat shock proteins [68]. The recognition of these molecules by pattern recognition receptors (PRR) such as Toll-like receptors (TLR) generates an innate immune response. Other chemoattractants secreted by activated platelets and endothelial cells, along with complement components, are also important for the recruitment of polymorphonuclear leukocytes, followed by monocytes/macrophages [66].

Recognition of the foreign structure is hastened by the adsorption of opsonins, mainly IgG and the C3b complement fragment at the surface of the biomaterial. Plasma-derived fibrinogen, fibronectin and vitronectin also promote the adhesion of neutrophils [65]. The interaction with these proteins is mediated by specific integrin receptors present on the surface of neutrophils and monocytes [67]. Phagocytosis, the primary function of neutrophils, is only possible in the case of powder, particulates or nanomaterials. Biomaterials larger than 5 microns are nonphagocytosable [65]. In this case, activated neutrophils try to degrade the biomaterial by releasing reactive oxygen species (ROS), enzymes and cytokines in an amount that is directly proportional to the particle size. This process is referred to as frustrated phagocytosis. It causes further tissue damage and erosion of the biomaterial, altering its function [65,67]. Generally, acute inflammation clears in a few days. However, the intensely pro-inflammatory local environment and the release by neutrophils of IL-8, monocyte chemoattractant protein (MCP)-1 and macrophage inflammatory protein (MIP)-1β explain the chronic inflammation that follows [66]. The persistence of neutrophils at the interface between the biomaterial and the tissue for more than 1 week is indicative of either infection or a leak of toxic degradation products from the biomaterial [65].

Researchers have investigated the effect of biomaterial surface adjustments meant to diminish the adsorption of proteins and the adhesion of leukocytes and to modulate the activity of neutrophils. Modified CD47-coated polyvinyl chloride surfaces, for instance, successfully decrease neutrophil recruitment and adhesion [69]. This was also observed with heparin-coated biomaterials [70]. Not only does heparin inhibit coagulation, but being negatively charged, it also immobilizes circulating cationic anaphylatoxins (C5a and C3a) [71]. Moreover, neutrophils that adhere to rough-surfaced biomaterials release ROS much faster than those that adhere to smooth-surfaced biomaterials [72].

### 4.3. Chronic Inflammation

Biomaterials may also generate chronic inflammation due to their specific chemical and physical attributes, their movement in the implant site and/or infection [36,65]. Chronic inflammation is generally restricted to the injured tissue. The inflammatory infiltrate consists of lymphocytes, monocytes and macrophages. The biomaterial’s nature and the type of proteins adsorbed at its surface greatly influence the duration of chronic inflammation, which ranges from days to months. As with acute inflammation, long-lasting infiltration of the implant site with monocytes and lymphocytes is associated with infection or toxic leachables [65,67].

Peripheral monocytes and macrophages respond to chemoattractant factors, such as complement components, TGF-β, platelet-derived growth factor (PDGF), platelet factor 4 (PF4), MCP-1, MIP-1α and MIP-1β, adhere to the provisional matrix through integrin receptors and become activated [66]. Activated macrophages are key cells of the chronic inflammatory response. Similar to the Th1/Th2 polarization of the immune response, macrophages also display two main phenotypes: the proinflammatory M1 phenotype and the anti-inflammatory, wound healing M2 phenotype [73]. Contrary to T lymphocytes, the transition from the M1 to M2 phenotype and vice versa commonly occurs, ensuring adequate host responses that may at any moment be suppressed in case they become excessive and harmful [74].

In the setting of chronic inflammatory reactions, the M1 phenotype is prevalent, mainly activated by interferon (IFN)-γ, lipopolysaccharide (LPS), tumor necrosis factor α (TNF-α) and granulocyte–macrophage colony-stimulating factor (GM-CSF) [67,75]. M1 macrophages secrete a wide array of active factors, such as lysosomal enzymes, chemotactic factors, arachidonate metabolites, ROS, nitric oxide, growth factors and pro-inflammatory cytokines [interleukin (IL)-1β, IL-6, IL-8, IL-12, IL-23 and TNF-α] [65,66,67]. M1 macrophages annihilate pathogens within the site of implantation and debride necrotic tissue. They show increased surface expression of major histocompatibility complex (MHC) II molecules and act as antigen-presenting cells. They also express the co-stimulatory molecule CD86. Through the release of IL-12 and IL-23, they activate T helper (Th) 1 and Th17 cells [67,75].

Unquestionably, the innate immune response plays a central role in the host’s response to biomaterials. Nevertheless, more and more evidence points to the implication of the adaptative immune response. Lymphocytes and primary CD4+ T cells are also part of the chronic inflammatory reaction to biomaterials. Th1 cells stimulate macrophages to adopt a pro-inflammatory phenotype, while Th2 cells, mainly through the production of IL-4 and IL-13, along with IL-10, IL-6, immune complexes, adenosine, glucocorticoids and macrophage colony-stimulating factor (M-CSF), activate the M2 macrophages that exert anti-inflammatory and pro-regenerative functions [66,67,75,76]. Th2 differentiation was shown to be essential for tissue repair after the implantation of biomaterials [77]. Although numerous cells of the innate response, such as neutrophils, mast cells, eosinophils, basophils and invariant natural killer T cells (iNKT), can produce IL-4 shortly after tissue injury, none is able to trigger Th2 differentiation by itself, suggesting a synergic action is necessary [76,78].

The properties of the biomaterial that influence the infiltration, adhesion and activation of macrophages have been the focus of intense research during the past decades. Macrophage adhesion is increased on hydrophobic and cationic biomaterial surfaces compared to hydrophilic and anionic surfaces [79]. Infiltration of the implantation site with macrophages and activation of macrophages was proven to be higher for biomaterials that contain corroding metals and hydroxyl and amino groups, as well as those with pores of approximately 30–40 µm or sharper edges, or in the case of stiff biomaterials implanted in loose tissues [80,81].

### 4.4. Formation of Granulation Tissue

Three to five days after the insertion of the biomaterial, a wound healing response unfolds [65]. As in the case of neutrophils attracted to the injured tissue in the initial phases of the inflammatory response, the proteins adsorbed on the biomaterial’s surface bind to integrin receptors present on the surface of macrophages and modulate their activity through various intracellular signaling cascades. As mentioned previously, soluble biomarkers, mainly IL-4, IL-13 and IL-10, activate the anti-inflammatory M2 macrophages [65,67]. In contrast to the M1 phenotype, these macrophages release IL-10 and ornithine that stimulate cell proliferation and promote the production of polyamine and collagen, with subsequent fibrosis [2,11,19]. They express numerous scavenger, mannose and galactose receptors—CD206, CD301 and CD163. M2 macrophages express TGF-β and αVβ8 integrin, participating in the polarization of the immune response to Th2 pathways and activating regulatory T cells [67,75,76].

The switch from the M1 to M2 phenotype and its timing are essential for tissue remodeling, leading to fibrosis or regeneration. Both prolonged M1 polarization and excessive activity of M2 macrophages may induce scarring and impede wound healing [67]. Once monocytes and macrophages switch to an anti-inflammatory phenotype, they release considerable amounts of PDGF, FGF, TGF-β, TGF-α/EGF and VEGF, as well as cytokines such as IL-1 and TNF-α [65]. All these stimulate the proliferation, growth and activation of fibroblasts, endothelial cells and numerous other cell types. They also trigger cell migration, differentiation and tissue remodeling, and promote neoangiogenesis, leading to the formation of granulation tissue. Therefore, chronic activation of M2 macrophages is associated with fibroblast hyperactivity. Fibroblasts excessively produce extracellular matrix components that accumulate at the biomaterial’s surface, as well as collagen, especially type III, which encloses the biomaterial in a fibrous capsule. Some of these fibroblasts differentiate in myofibroblasts and induce wound contraction [65,76].

### 4.5. Foreign Body Reaction

Subsequently, the host response to the presence of non-degradable biomaterials, especially synthetic or metallic biomaterials, may take the form of a foreign body reaction (FBR), consisting of components of the granulation tissue, i.e., macrophages, fibroblasts, blood vessels in different proportions and multinucleated giant cells [65,76].

Biological scaffolds, on the other hand, rarely generate FBR. They have been shown to polarize macrophages to a pro-regenerative phenotype characterized by CD206 expression [82], most probably due to the activation of a Th2-mediated immune response [76].

Foreign body giant cells (FBGC) form as a result of the fusion of macrophages around the foreign body. The fusion requires the expression of several molecules at the surface of macrophages, principally mannose receptors DC-stamp, CD206, CD13, CD44, CD47, E-cadherin and galectin-3 [66,75], under the influence of IL4, but also IL-13, IL-3 and MMP9 [75,83]. During the early stages of FBR, FBGC secrete IL-1α, IL-6, IL-8 and TNF-α, and they later release IL-10, TGF-β and MCP-1 [67]. Taking all these into account, the exact phenotype of the macrophages that form FBGC has not been elucidated as they share features of both M1 and M2 phenotypes [84]. Some authors consider FBGC a distinct macrophage type [83].

The intensity of FBR is also influenced by the biomaterial’s properties. Anionic and non-ionic hydrophilic polyacrylamide/polyacrylic acid surfaces are associated with less FBGC formation than cationic and either hydrophilic or hydrophobic surfaces [79].

In the case of smooth-surfaced biomaterials (e.g., breast prostheses), the foreign body reaction is usually composed of a mono- or bilayer of macrophages; the FBR induced by rough-surfaced biomaterials (e.g., Dacron vascular prostheses) also features foreign body giant cells. The reaction to fabric materials incorporates foreign body giant cells, macrophages and varying degrees of granulation tissue [65].

In addition, the form and surface-to-volume ratio of the biomaterial greatly influences the proportion of the elements of the FBR. Fabrics, porous or particulate materials and microspheres, all of which are characterized by a high surface-to-volume ratio, induce a FBR mainly composed of macrophages and foreign body giant cells. On the other hand, fibrosis is a substantial part of the FBR generated by smooth-surfaced biomaterials that become encapsulated and therefore isolated from the surrounding tissue [65].

FBGC formation is more intense with polylactic-co-glycolic acid microspheres of approximately 30 µm than microspheres of approximately 6 µm [85]. Contrarily, spherical biomaterials with a diameter larger than 1.5 mm seem to induce less fibrosis than smaller spheric biomaterials or biomaterials of a different shape, irrespective of their composition [86].

The foreign body reaction may persist indefinitely, often for the entire lifespan of the biomaterial [36,65]. It is not clear if the cellular components remain active throughout this period. FBGC release ROS, enzymes and acids that degrade the biomaterial. Certain surface chemical properties of the biomaterial induce the apoptosis of harmful adherent macrophages in order to protect both the biomaterial and the tissue of implantation. Nevertheless, macrophages may evade the process of apoptosis and coalesce into giant cells. Altogether, the FBR negatively impacts biomaterial durability, integration and performance, as well as the tissue’s structure and function [65,67].

### 4.6. Fibrosis/Fibrous Encapsulation

The final phase of the host’s response to biomaterials is represented either by the regeneration of the injured tissue by proliferative parenchymal cells after the degradation of the biomaterial or by the replacement of the connective tissue by fibrosis and fibrous encapsulation of the biomaterial. The outcome depends on a series of factors, among which are the degree of tissue injury upon biomaterial implantation, the proliferative capacity of the cells of that certain tissue and the preservation of the tissular structure after the insertion of the biomaterial [65].

In tissues in which the predominant cellular population has no replicative potential, such as nerve cells and cardiac muscle cells, chronic inflammation invariably leads to fibrosis. Although this may also be the end stage of the host reaction in tissues composed of cells that maintain the capacity to proliferate, they may also undergo regeneration and, finally, restoration of their normal structure. This also requires the maintenance of the tissue’s framework after the implantation of the biomaterial, as tissue repair is not possible if the supporting stroma for proliferative cells is destroyed. Of utmost importance for effective wound healing are local factors, such as tissue type, vascularization, risk of infection and systemic factors, among which are the state of nutrition, pre-existing conditions and chronic medication [65].

Tissue remodeling is greatly influenced by the activity of T cells. As mentioned above, Th2 differentiation and IL-4 release promote tissue regeneration processes. Still, other cytokines produced by Th2 cells may have detrimental effects. Persistent secretion of IL-13 induces excessive ECM deposition and promotes fibrosis [60,87]. Macrophages also sustain prolonged IL-13 production during granulomatous inflammatory reactions, favoring fibrosis [88]. M2 macrophages and other activated cells also release PDGF, VEGF, TGF-β and galactin-3, which are potent stimulators of fibrosis and angiogenesis [38,66]. Moreover, macrophages and endothelial cells secrete matrix metalloproteinases (MMP) that participate in ECM remodeling [66].

In addition, Th17 cells, primarily through the release of IL17A, stimulate granulocyte–macrophage colony stimulating factor (GM-CSF) production, enhancing polarization of macrophages towards a pro-fibrotic phenotype [89].

Interestingly, B cells have also been found in the fibrous capsule formed around the biomaterial, concomitantly with an increase in activated myofibroblasts. B cells are attracted to the implantation site mainly by CXCL13 secreted by macrophages. It seems plausible that the delayed B cell response contributes to the recruitment of myofibroblasts, enhancing fibrosis. B cells promote fibrosis through the release of cytokines and not antibodies, the mechanism resembling the innate immune response [90].

Once again, the shape, size, surface and composition of the biomaterial greatly influence the formation of the fibrous capsule. A thinner fibrous capsule and better wound healing were observed when biomaterials with increased porosity, thin plane or circular biomaterials larger than 1.5 mm microspheres, polyethylene fibers less than 6 µm in diameter or biomaterials composed of polyurethane with silicone and polyethylene oxide were used [66]. Gelatin-based hydrogels or hydrogels containing polycarboxybetaine methacrylate generate less fibrosis than poly-2-hydroxyethyl methacrylate [66]. On the other hand, amino and hydroxyl groups on hydrophilic surfaces and carboxyl groups on hydrophobic surfaces induce much thicker fibrous capsules [91,92,93].

Perfect healing, with the restoration of the normal structure and function of the injured tissue, is rarely achieved. As current knowledge is limited with regard to the means of controlling the reactions triggered by the implantation of biomaterials, studies on the adequate modulation of the host inflammatory response, maintaining the balance between innate and adaptive immune responses, stimulation of Th2 and M2 pathways, use of growth factors, promotion of cellular proliferation, incorporation of biological signals able to stimulate tissue regeneration, optimization of wound healing, and prevention of fibrous encapsulation of biomaterials are warranted.

## 5. Conclusions

Biocompatibility is thus a primary characteristic of wound dressing intended for use in living tissues. Thus, it must maintain a perfect balance between promoting healing and preventing infection, but without being carcinogenic or immunogenic (these can have important harmful effects).

Although the polymers from which they are made show a great adaptability, it is important, in addition to their efficiency and immune tolerance, to achieve the subsequent efficient elimination of biomaterials from the body, after they have fulfilled their function. Thus, biomaterials containing biodegradable bioparticles that degrade and are eliminated from the body after fulfilling their function are preferred, unlike non-biodegradable ones that tend to accumulate in key organs (liver and spleen), potentially having long-term effects.

In conclusion, a dressing can be considered ideal when it fulfills a series of characteristics, such as it maintains moisture and absorbs exudate from the wound, but it is permeable to water and gas; it is biocompatible, not determining the body’s immune reactions; it does not cause allergic reactions and it is produced from biodegradable materials that do not accumulate. Last but not least, it should be easy to use by the patient and practitioner and have a favorable cost-effectiveness ratio. The multitude of these characteristics makes it difficult to find an ideal wound dressing, but a detailed knowledge of these variables is necessary in order to choose the best variant adapted to the patient and the type of injury.

## Data Availability

Not applicable.
